# The Prevalence, Size, and Anatomic Location of Cartilage and Osteochondral Lesions in Athletes With an Acute Ligamentous Ankle Injury

**DOI:** 10.1177/03635465251344187

**Published:** 2025-06-12

**Authors:** Thomas P.A. Baltes, Feriel Dalansi, Maryam R. Al-Naimi, Marcelo Bordalo, Louis Holtzhausen, Rod Whiteley, Marco Cardinale, Pieter D’Hooghe, Gino M.M.J. Kerkhoffs, Johannes L. Tol

**Affiliations:** † Research Department, Aspetar Orthopaedic and Sports Medicine Hospital, Doha, Qatar; ‡ Department of Orthopaedic Surgery and Sports Medicine, Amsterdam UMC, University of Amsterdam, Amsterdam Movement Sciences, Amsterdam, The Netherlands; § Academic Center for Evidence-Based Sports Medicine (ACES), Amsterdam UMC, Amsterdam Movement Sciences, Amsterdam, The Netherlands; ‖ Amsterdam Collaboration for Health and Safety in Sports (ACHSS), AMC/VUmc IOC Research Center, Amsterdam, The Netherlands; ¶ Department of Radiology, Aspetar Orthopaedic and Sports Medicine Hospital, Doha, Qatar; # Department of Sports Medicine, Aspetar Orthopaedic and Sports Medicine Hospital, Doha, Qatar; ** Section Sports Medicine, Faculty of Health Sciences, University of Pretoria, Pretoria, South Africa; †† Department of Exercise and Sports Science, University of the Free State, Bloemfontein, South Africa; ‡‡ Department of Rehabilitation, Aspetar Orthopaedic and Sports Medicine Hospital, Doha, Qatar; §§ Institute of Sport, Exercise and Health, University College London, London, UK; ‖ ‖ Department of Orthopaedic Surgery, Aspetar Orthopaedic and Sports Medicine Hospital, Doha, Qatar; Investigation performed at Aspetar Orthopaedic and Sports Medicine Hospital, Doha, Qatar

**Keywords:** ankle sprain, cartilage injury, osteochondral lesions, ankle ligaments, magnetic resonance imaging

## Abstract

**Background:**

In athletes with an acute ligamentous ankle injury, cartilage and osteochondral lesions ([O]CLs) have been reported in 8% using 1.5-T magnetic resonance imaging (MRI). Visualization of cartilage injuries improves with the use of higher field strengths.

**Purpose:**

To evaluate the prevalence, size, and anatomic location of (O)CLs in athletes with an acute ligamentous ankle injury using 3-T MRI, as well as to determine the association of (O)CLs with injury of (1) the lateral ankle ligaments and (2) anterior syndesmosis.

**Study Design:**

Cohort study; Level of evidence, 3.

**Methods:**

For this prospective cohort study, all acute ligamentous ankle injuries in athletes (≥18 years of age) evaluated in the outpatient department of a specialized orthopaedic and sports medicine hospital within 7 days after injury were assed for eligibility. Acute ankle injuries were excluded if 3-T MRI could not be obtained within 10 days after injury or if imaging demonstrated a frank fracture. A musculoskeletal radiologist assessed MRI scans for the presence, location, and size of (O)CLs. Morphology was graded using the modified Berndt and Harty score, Griffith MRI score, and International Cartilage Regeneration & Joint Preservation Society score. In addition, injuries of the lateral ankle ligaments and anterior syndesmosis were graded. A multivariate logistic regression analysis was performed to evaluate the association between (O)CLs and injury of the (1) lateral ankle ligaments and (2) anterior syndesmosis.

**Results:**

Between September 2016 and February 2020, 171 acute ankle injuries in 166 athletes were included in this study. The overall prevalence of (O)CLs was 14%. (O)CLs of the talus and tibia were observed in 24 (14%) and 9 (5%) acute ankle injuries, respectively. Of 33 (O)CLs, 28 (85%) were classified as cartilage lesions. Lateral ligament injury was observed in 73% of acute ankle injuries, and anterior syndesmosis injury in 38%. Multivariate logistic regression analysis did not show significantly higher odds of (O)CLs in the presence of anterior syndesmosis injury (OR, 2.16; 95% CI, 0.90-5.16).

**Conclusion:**

In athletes with an acute ligamentous ankle injury, a prevalence for (O)CLs of 14% was established using 3-T MRI. The majority were cartilage lesions. No statistically significant association was established between (O)CLs and lateral ligament or syndesmosis injury was established.

Concomitant pathology of the ankle is frequently observed after acute ligamentous ankle injuries.^17,21^ Concomitant pathology may include (avulsion) fractures, ligamentous injuries (eg, syndesmosis), tendon injuries, and osteochondral lesions. Osteochondral lesions are hypothesized to be part of a degenerative cascade, beginning with (1) partial-thickness cartilage surface lesions, then progressing to (2) osteochondral lesions and finally (3) end-stage osteoarthritis.^13^ Early detection of cartilage and osteochondral lesions ([O]CLs) might help mitigate the risk of osteoarthritis. Understanding the prevalence (including size and anatomic location) of (O)CLs could aid in the identification of athletes at risk of developing osteoarthritis.

In a recent meta-analysis, the prevalence of (O)CLs in chronic ankle instability was investigated.^27^ In this meta-analysis of 12 studies (including 2145 patients), a prevalence of 32% was reported using intraoperative findings. In athletes with an acute ligamentous ankle injury, one previous study has evaluated the prevalence of osteochondral lesions only.^21^ In this cohort study of 261 athletes who underwent 1.5-T magnetic resonance imaging (MRI) after an acute ligamentous ankle injury, a prevalence of 8% was reported for osteochondral lesions. As visualization of cartilage lesions improves with the use of higher field strengths, the prevalence of (O)CLs could be higher than the previously reported 8% for osteochondral lesions only.^11^ A prospective cohort study using 3-T MRI in athletes with an acute ligamentous ankle injury is therefore warranted.^6^

For athletes with syndesmosis injuries, the prevalence of (O)CLs was also reported in a recent meta-analysis.^12^ In this meta-analysis of 9 studies (including 402 syndesmotic injuries), a prevalence of 21% was reported using MRI and arthroscopic findings. This is higher than the previously reported prevalence of 8% in athletes with an acute ligamentous ankle injury.^21^ Therefore, exploring the association between (O)CLs and lateral ligament and anterior syndesmosis injuries could help decide which athletes need referral for additional MRI.

Considering the lack of consensus on the prevalence of (O)CLs and the potential for 3-T MRI to improve detection, the primary aim of this study was to describe the prevalence, size, and anatomic location of (O)CLs in athletes with an acute ligamentous ankle injury. The secondary aim was to determine the association of (O)CLs with injury of the (1) lateral ankle ligaments and (2) anterior syndesmosis.

## Methods

### Participants

Data used in this cross-sectional study were collected in the context of a prospective cohort study on the diagnosis and outcome of acute ligamentous ankle injuries in athletes.^1-5^ For this study, all acute ligamentous ankle injuries in athletes ≥18 years of age evaluated in the outpatient department of a specialized orthopaedic and sports medicine hospital within 7 days after injury were assessed for eligibility. Acute ankle injuries were excluded if 3-T MRI scans could not be obtained within 10 days after injury or if imaging demonstrated a frank fracture. Written informed consent was obtained from all athletes at the time of inclusion. Ethics approval was obtained from the institutional review board (Anti-Doping Lab Qatar; IRB No. F2016000153).

### Imaging

All athletes underwent 3-T MRI (GE Discovery; GE HealthCare) with an 8-channel receive-only foot and ankle array (Invivo; Philips Healthcare). The imaging protocol has been described previously.^3^

### Grading of Ligamentous Injury

The MR scans (≤10 days after injury) were evaluated for ligamentous ankle injuries by a musculoskeletal radiologist (M.R.A.N.) with 10 years of experience. The radiologist graded the ligamentous lesions of the lateral ankle ligaments (anterior talofibular ligament [ATFL], calcaneofibular ligament [CFL], and posterior talofibular ligament [PTFL]) and syndesmotic ligaments (anterior inferior tibiofibular ligament [AITFL], interosseous ligament [IOL], interosseous membrane [IOM], and posterior inferior tibiofibular ligament [PITFL]) according the Schneck grading system^22^: normal (grade 0), low-grade sprain (grade 1: periligamentous high signal/edema on proton density–weighted sequences and no discontinuity of fibers), partial discontinuity (grade 2: partial discontinuity but preserved remnant fibers), and complete discontinuity (grade 3).

### Grading of (O)CLs

The presence of (O)CLs of the talus and tibia was evaluated by a senior musculoskeletal radiologist (M.B.) with 24 years of experience. A previous study demonstrated almost perfect interrater reliability for the detection of osteochondral lesions in the acute setting.^21^ Cartilage lesions were defined as injury of the superficial cartilage with or without subsequent involvement of the subchondral bone.^8,13^ Osteochondral lesions were defined as compressed areas of trabecular bone with or without damage of the overlying cartilage (transchondral fracture) or an avulsion of an osteocartilaginous flake.^7^ When an (O)CL was observed, the size (in mm) was measured in 3 directions (anteroposterior, mediolateral, and superoinferior) (interobserver reliability κ = 0.87).^25^ The anatomic location was recorded using the 9-grid scales developed by Elias et al^14,15^ (interobserver reliability κ = 0.55).^18^ Cartilage lesions were graded using the International Cartilage Regeneration & Joint Preservation Society (ICRS) score.^9^ Morphology of osteochondral lesions was graded using the modified Berndt and Harty score, Griffith MRI score, and ICRS score^9,16,23^ (see Appendices 1-3, available in the online version of this article). When multiple lesions were observed, the largest measurement was reported.

### Statistical Analysis

Demographics, prevalence, size, and anatomic location of the (O)CLs were reported using descriptive statistics. To determine the association between the presence of (O)CLs and (1) lateral ligament and (2) anterior syndesmosis injury, a multivariate logistic regression analysis (enter method) was performed. For this analysis, lateral ligament injury was defined as a partial or complete discontinuity of the anterior talofibular ligament and/or calcaneofibular ligament. Injury of the syndesmosis ligaments was defined as a partial or complete tear of the AITFL. Athletes were considered to have an (O)CL if they had an ICRS grade ≥1A (cartilage lesion) or a modified Berndt and Harty grade ≥1 (osteochondral lesion). Statistical analysis was performed using SPSS software (Version 27; IBM Corp).

## Results

**Figure 1.**
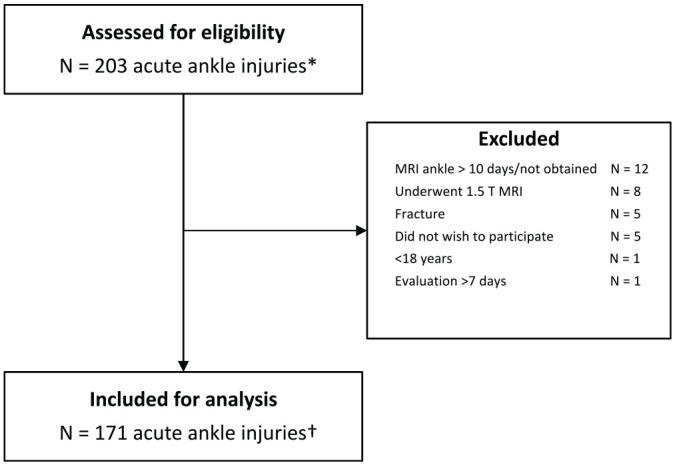
Flowchart for study inclusion. ^*^In 194 athletes. ^†^In 166 athletes. MRI, magnetic resonance imaging.

A total of 171 acute ankle injuries in 166 athletes were included in this study (Figure 1). Of the 171 included acute ankle injuries, 4 were subsequent contralateral ankle injuries and 1 was a reinjury (>1 year). Patient characteristics are shown in Table 1. The details of athletes referred for surgery (≤1 year after injury) are provided in Appendix 4 (available online).

**Table 1:** Description of Included Ankle Injuries*^a^*

	Value
Time to clinical evaluation, days	2 (1-3)
Time to MRI, days	3 (2-5)
Age at time of injury, years	24 (20-28)
Side, right	85 (50)
History of ipsilateral ankle sprains	43 (25)
Sports	
Soccer	75 (44)
Volleyball	26 (15)
Futsal	15 (9)
Basketball	12 (7)
Handball	12 (7)
Athletics	5 (3)
Other sports	26 (15)

a Data are presented as n (%) or median (IQR). MRI, magnetic resonance imaging.

### Overall Prevalence of (O)CLs

**Figure 2.**
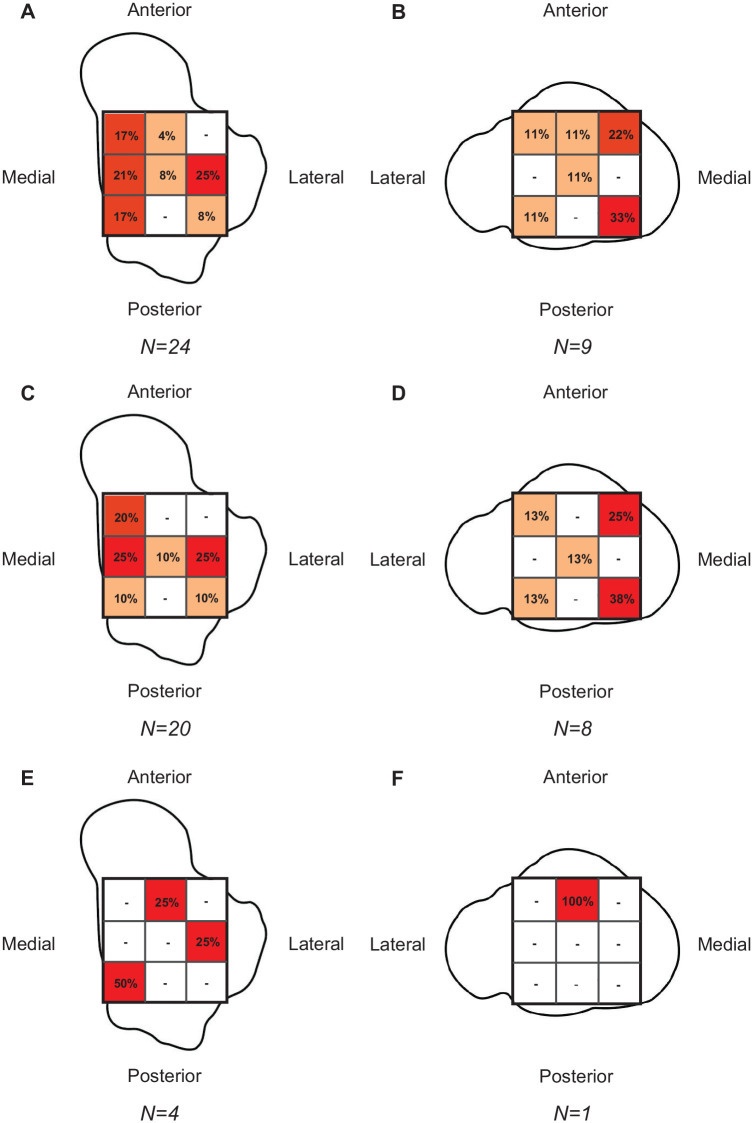
Distribution of cartilage and osteochondral lesions ([O]CLs) expressed as a percentage of total lesions present on the talus or tibia. Distribution of (O)CLs on (A) talus and (B) tibia. Distribution of cartilage lesions on (C) talus and (D) tibia. Distribution of osteochondral lesions on (E) talus and (F) tibia.

The overall prevalence of (O)CLs was 14% (24/171) (Table 2). (O)CLs of the talus and tibia were observed in 24 (14%) and 9 (5%) acute ankle injuries, respectively (Figure 2, A and B). (O)CLs were observed in 6 of 77 (8%) acute ankle injuries with lateral ligament injury, 2 of 18 (11%) acute ankle injuries with syndesmosis injury, and 11 of 47 (23%) acute ankle injuries with combined injury of the lateral and anterior syndesmosis ligaments.

**Table 2:** Size, Location, and Grading of Cartilage and Osteochondral Lesions According to the Modified Berndt and Harty, Griffith, and ICRS Classification Systems*^a^*

ID	Type	Location	Size AP, mm	Size ML, mm	Depth, mm	Modified B&H Grade	Griffith Grade	ICRS Grade
Talus								
2	Cartilage	Central	4	1	1	NA	NA	1B
8	Cartilage	Anteromedial	5	3	1	NA	NA	2
9	Cartilage	Posterolateral	4	3	1	NA	NA	3A
16	Osteochondral	Posteromedial	16	11	1	2	4B	4
25	Cartilage	Lateral	6	2	1	NA	NA	3B
28	Cartilage	Lateral	8	6	1	NA	NA	4
38	Cartilage	Medial	1	1	1	NA	NA	3C
39	Cartilage	Posterolateral	15	12	1	NA	NA	3C
68	Cartilage	Anteromedial	2	3	1	NA	NA	3A
74	Cartilage	Medial	10	7	2	NA	NA	4
85	Cartilage	Posteromedial	10	9	1	NA	NA	3A
86	Cartilage	Posterolateral	4	5	1	NA	NA	3C
93	Osteochondral	Anterior	3	3	1	1	2B	4
94	Cartilage	Medial	4	2	1	NA	NA	4
112	Cartilage	Medial	2	3	1	NA	NA	3D
116	Osteochondral	Posteromedial	4	4	1	1	2B	4
120	Cartilage	Central	19	3	1	NA	NA	4
130	Osteochondral	Lateral	11	8	3	2	3B	4
145	Cartilage	Lateral	8	5	2	NA	NA	4
147	Cartilage	Anteromedial	8	9	1	NA	NA	2
153	Cartilage	Anteromedial	5	6	1	NA	NA	3C
157	Cartilage	Lateral	8	3	1	NA	NA	4
164	Cartilage	Medial	5	3	2	NA	NA	3A
194	Cartilage	Lateral	4	2	1	NA	NA	3C
Tibia								
16	Cartilage	Posteromedial	13	7	1	NA	NA	4
28	Cartilage	Anterolateral	4	4	1	NA	NA	3B
39	Cartilage	Posterolateral	8	8	1	NA	NA	3C
68	Cartilage	Anteromedial	3	1	1	NA	NA	2
85	Cartilage	Posteromedial	1	1	1	NA	NA	3C
93	Osteochondral	Anterior	5	9	1	1	2B	4
120	Cartilage	Central	12	9	1	NA	NA	4
147	Cartilage	Anteromedial	4	5	1	NA	NA	4
164	Cartilage	Posteromedial	2	3	1	NA	NA	1B

a Description of included acute ankle injuries with cartilage and osteochondral lesions. AP, anteroposterior; B&H, Berndt and Harty; ICRS, International Cartilage Regeneration & Joint Preservation Society; ML, mediolateral; NA, not applicable.

### Prevalence, Size, and Location of Cartilage Lesions

The prevalence of cartilage lesions was 12% (20/171) (Table 2). Cartilage lesions of the talus and tibia were observed in 20 (12%) and 8 (5%) acute ankle injuries, respectively. The median size of the talar cartilage lesions was 22 mm^3^ (IQR, 8-68 mm^3^). Talar cartilage lesions were primarily located lateral (25%), medial (25%), and anteromedial (20%). Tibial cartilage lesions were a median size of 18 mm^3^ (IQR 4-84 mm^3^) and primarily located on the posteromedial (37.5%) and anteromedial (25%) aspects (Figure 2, C and D).

### Prevalence, Size, and Location of Osteochondral Lesions

The prevalence of osteochondral lesions was 2% (4/171) (Table 2). Osteochondral lesions of the talus and tibia were observed in 4 (2%) and 1 (1%) acute ankle injuries, respectively. The median size of talar osteochondral lesions was 96 mm^3^ (IQR, 11-242 mm^3^). Talar osteochondral lesions were located on the posteromedial (50%), lateral (25%), and anterior (25%) aspects (Figure 2, E and F).

### Association Between (O)CLs and Ligamentous Injury

Injury of the lateral ankle ligaments was observed in 73% of acute ankle injuries. Injury of the anterior syndesmosis was observed in 38% of acute ankle injuries. Results of the analysis for the association between (O)CLs and location of ligamentous injury are reported in Table 3. Multivariate logistic regression analysis demonstrated nonstatistically significant higher odds of (O)CLs in the presence of syndesmosis injury (OR, 2.16; 95% CI, 0.90-5.16; *P* = .08) compared with lateral ligament injury.

**Table 3:** Multivariate Logistic Regression Analysis for the Association Between (O)CLs and Ligamentous Injury Location*^a^*

	N	OR (95% CI)	SE	*P* Value
Cartilage injury (including osteochondral lesions)				
Lateral ligament injury	124	0.91 (0.35-2.38)	0.49	.85
Syndesmosis injury	65	2.16 (0.90-5.16)	0.45	.08

a The odds ratio for the independent predictor associated with cartilage and osteochondral lesions ([O]CLs) is presented. A lateral ligament injury was defined as a partial or complete discontinuity of the anterior talofibular ligament and/or calcaneofibular ligament. A syndesmosis injury was defined as a partial or complete discontinuity of the anterior inferior tibiofibular ligament. Odds ratio (OR) are presented with corresponding 95% CI and standard error (SE).

## Discussion

The most important finding in this prospective cohort study of 171 acute ankle injuries (166 athletes) is the relatively high prevalence of (O)CLs (14%) as determined by MRI. (O)CLs of the talus and tibia were observed in 24 (14%) and 9 (5%) acute ligamentous ankle injuries, respectively. The majority (85%) of (O)CLs were classified as cartilage lesions. Syndesmosis injury demonstrated nonstatistically significantly higher odds of (O)CLs (OR, 2.16; 95% CI, 0.90-5.16) compared with lateral ligament injury. The finding of this study suggests that (O)CLs are more prevalent in acute ligamentous ankle injuries than previously reported.

### Comparison With Previous Literature: Prevalence

The overall prevalence of (O)CLs in this study was 14%. A previous study of 261 athletes with an acute ligamentous ankle injury, from the same specialized orthopaedic and sports medicine hospital, established a prevalence of 8% using 1.5-T MRI.^21^ A possible explanation for the higher prevalence in the current study is the use of high-field-strength (3 T) MRI, which improves the visualization of superficial cartilage lesions. These findings are consistent with the literature on the use of MRI for the detection of cartilage injuries of the knee. In a meta-analysis of 16 studies (including 1886 patients), 3-T MRI demonstrated higher diagnostic accuracy for the detection of cartilage lesions of the knee in comparison with 1.5-T MRI.^11^

In patients with a syndesmosis injury, a recent meta-analysis reported a prevalence of (O)CLs in up to 21% using both MRI and arthroscopic findings.^12^ Despite the hypothesis of the current study, no statistically significant association between (O)CLs and syndesmosis injury was established. A possible explanation might be that in the current study partial and/or complete discontinuity of the anterior syndesmosis (AITFL) was considered disease positive. Thus, clinically stable syndesmosis injuries (West Point grade ≤IIA) were included, whereas in the literature most studies include patients with unstable syndesmosis injuries requiring surgical stabilization (West Point grade ≥IIB).^10,12^ Future prospective cohort studies should therefore aim to investigate the correlation of clinical stability (stress test assessment) with the prevalence of (O)CLs.

### Comparison With Previous Literature: Anatomic Location

The primary anatomic location of (O)CLs was the talus (14%). (O)CLs of the tibia were observed in 5% and only in the presence of (O)CLs of the talus. (O)CLs of the talus were located on the medial and posteromedial aspects in 38% and the lateral aspect in 25%. These findings are in line with the findings of a recent meta-analysis on the distribution of (O)CLs of the talus. In that study, the posteromedial (28%) and medial (31%) zones of the talus had the highest prevalence of (O)CLs.^24^ The distribution pattern of (O)CLs in the current study suggests that a subset of athletes sustained an inversion injury of the ankle, with either a plantarflexed ankle (medial talar [O]CLs) or dorsiflexed ankle (lateral talar [O]CLs).^7^ Previous studies reporting on the anatomic location of (O)CLs in patients with isolated syndesmosis injuries have reported the highest prevalence at the anterolateral and lateral aspects of the talus.^20,26^ Although in the current study a prevalence of 25% was reported for the lateral aspect of the talus, no (O)CLs were observed at the anterolateral aspect of the talus. The findings in the current study warrant further research to elucidate the association between trauma mechanism, injured ligamentous complexes, and location of (O)CLs.

### Comparison With Previous Literature: Grading of (O)CLs

Various grading systems have been reported to describe the morphology of (O)CLs.^25^ In a previous study cohort study of 261 athletes who underwent 1.5-T MRI after an acute ligamentous ankle injury, osteochondral lesions were graded on a 6-grade scale (0 = normal, 1 = small contusion, 2 = large contusion, 3 = acute osteochondral lesion with intact cartilage, 4 = acute osteochondral lesion with cartilage injury, and 5 = chronic osteochondral lesion).^21^ In the current study, we used the modified Berndt and Harty classification (based on radiographic findings), as it is more commonly used in clinical practice.^23^ In the current literature, later adaptations of this classification (Hepple and Anderson classification systems) are most commonly used to describe MRI-based (O)CL morphology.^25^ The Griffith MRI grading system was incorporated in this study as it appreciates the presence of cartilage fractures.^16^ Finally, the ICRS classification (based on arthroscopic findings) was added as it enables description of injuries limited to the superficial cartilage.^9^ Despite various (O)CL morphology classifications having been described, validity and reliability studies are lacking.^25^ Future studies should therefore aim to determine the diagnostic reliability and cross-modal validity.

### Development of Osteoarthritis

Osteoarthritis is characterized by the degradation of joint cartilage and underlying bone, leading to pain, swelling, and reduced motion. In acute ligamentous ankle injuries, (O)CLs are hypothesized to be either (1) asymptomatic superficial cartilage lesions that will remain asymptomatic; (2) posttraumatic cartilage cracks that, with involvement of the subchondral bone, might become symptomatic; and (3) large acute symptomatic lesions with involvement of the subchondral bone plate. It is important to note that not all cartilage injuries become symptomatic and lead to end-stage ankle osteoarthritis. However, no prognostic factors to determine which (O)CLs will become symptomatic or progress to end-stage ankle osteoarthritis have been identified. In the current study, the majority of talar (58%) and tibial (56%) (O)CLs were classified as superficial cartilage injuries (ICRS grades 1-3), supporting the theory that a subset of superficial cartilage injuries remains asymptomatic. However, a large prospective cohort study identifying prognostic factors for the development of symptomatic (O)CLs and end-stage osteoarthritis in athletes with an acute ligamentous ankle injury is warranted.

### Strengths and Limitations

The main strength of this study is the use of high-field-strength MRI (3-T) in a large prospective cohort of athletes with acute ligamentous ankle injuries to determine the prevalence of (O)CLs. Despite the use of high-field-strength MRI, the diagnostic accuracy of (O)CLs remains inferior to the use of (needle) arthroscopy.^26^ Furthermore, selection bias may have occurred, as athletes with a mild acute ligamentous ankle injury might not have presented for clinical and radiological evaluation and completed rehabilitation at their club. Finally, only the ICRS grading system was applied to cartilage lesions. This is because of the composition of the modified Berndt and Harty and Griffith grading systems (bone marrow edema precedes transchondral fracture), which is consistent with the definition used for osteochondral lesions. The ICRS classification system is more suitable to grade cartilage injuries (cartilage injury precedes involvement of the subchondral bone).

### Clinical Implications

The most important finding in this study is that 3-T MRI–determined (O)CLs are more common than previously reported. The study should raise awareness among clinicians of the prevalence of concomitant (O)CLs in athletes with an acute ligamentous ankle injury. In clinical practice, early diagnosis and appropriate management of ligamentous injuries (eg, unstable syndesmosis injury) using physical examination or ultrasound should be paramount.^2,4^ If physical rehabilitation for an athlete fails despite adequate treatment, referral for MRI to rule out (O)CLs should be considered. When an (O)CL is present, MRI may provide valuable information on size, location, and grading of the (O)CL and can be used to guide treatment.^19^ Future large prospective cohort studies should aim to identify clinical predictors (eg, age, history of ipsilateral ankle sprain, and stress instability) associated with the presence of (O)CLs.

## Conclusion

In athletes undergoing 3-T MRI for an acute ligamentous ankle injury, the prevalence of (O)CLs is 14%. The majority of these injuries were cartilage lesions. The findings of this study suggest that (O)CLs are more prevalent in athletes with an acute ligamentous ankle injury than reported previously in studies utilizing 1.5-T MRI.

## Supplemental Material

sj-pdf-1-ajs-10.1177_03635465251344187 – Supplemental material for The Prevalence, Size, and Anatomic Location of Cartilage and Osteochondral Lesions in Athletes With an Acute Ligamentous Ankle Injury Supplemental material, sj-pdf-1-ajs-10.1177_03635465251344187 for The Prevalence, Size, and Anatomic Location of Cartilage and Osteochondral Lesions in Athletes With an Acute Ligamentous Ankle Injury by Thomas P.A. Baltes, Feriel Dalansi, Maryam R. Al-Naimi, Marcelo Bordalo, Louis Holtzhausen, Rod Whiteley, Marco Cardinale, Pieter D’Hooghe, Gino M.M.J. Kerkhoffs and Johannes L. Tol in The American Journal of Sports Medicine
