# Supplementary material for: The Prevalence, Size, and Anatomic Location of Cartilage and Osteochondral Lesions in Athletes With an Acute Ligamentous Ankle Injury
Source: Am J Sports Med. 2025 Jun 12;53(9):2173–80. doi: 10.1177/03635465251344187 (PMC12235055; doi:10.1177/03635465251344187)
Supplement: sj-pdf-1-ajs-10.1177_03635465251344187 – Supplemental material for The Prevalence, Size, and Anatomic Location of Cartilage and Osteochondral Lesions in Athletes With an Acute Ligamentous Ankle Injury [file sj-pdf-1-ajs-10.1177_03635465251344187.pdf]

Appendix 1 Modified Berndt & Harty

Grade	Description
1	Subchondral bone compression; marrow edema
2	Chip avulsed, but attached
3	Detached chip, but undisplaced
4	Detached and displaced chip
5	Talar cyst, with adjacent edema of the talar body

Appendix 2 Griffith MRI-score

Grade	Description
1a	Bone marrow change (edema, cystic change) with no collapse of subchondral bone area, no osteochondral junction separation and intact cartilage.
1b	Grade 1a + cartilage fracture present.
2a	Variable collapse of subchondral bone area with osteochondral separation though intact Cartilage.
2b	Grade 2a + cartilage fracture present. This is an unstable lesion with level of instability related to extent of cartilage fracture.
3a	Variable collapse of subchondral bone area with no osteochondral separation +/- variable cartilage hypertrophy
3b	Grade 3a + cartilage fracture present
4a	Separation within or at edge of bone component with intact overlying cartilage
4b	Grade 4a + cartilage fracture present. This is an unstable lesion with level of instability related to extent of cartilage fracture
5	Complete detachment of osteochondral lesion. This is an unstable lesion.

Appendix 3 ICRS-classification

Grade	Description
0	Macroscopically normal cartilage without notable defects
1A	Intact surface but fibrillation and/or slight softening is present
1B	ICRS 1a + superficial lacerations and fissures are found
2	Defects that extend deeper but involve <50% of the cartilage thickness
3A	Deep defects that extend through >50% of the cartilage depth but not to the calcified layer
3B	Deep defects that extend through >50% of the cartilage depth to the calcified layer
3C	Defects that extend down to but not through the subchondral bone plate
3D	Blisters
4	Cartilage defects that extend into the subchondral bone

Appendix 4 Athletes referred for surgery

ID	MRI lateral	MRI syndesmosis	MRI (O)CLs	Type of surgery
#8	ATFL gr 1; CFL gr 1	AITFL/IOL gr 3; IOM gr 2; PITFL gr 1	Cartilage ICRS grade 2	Syndesmotic fixation
#24	-	AITFL/IOL gr 3; IOM gr 2; PITFL gr 1	-	Syndesmotic fixation
#34	ATFL gr 3; CFL gr 3	AITFL/IOL gr 3; IOM gr 2	-	Syndesmotic fixation
#53	ATFL gr 3; CFL gr 2	AITFL gr 1	-	Broström + Deltoid repair
#57	ATFL gr 3; CFL gr 3	AITFL gr 3; IOL gr 3; IOM gr 2	-	Syndesmotic fixation + Broström
#64	ATFL gr 2; CFL gr 3	AITFL gr 1	-	Broström
#67	ATFL gr 3; CFL gr 3	AITFL gr 1	-	Broström
#71	ATFL gr 3; CFL gr 3	AITFL/IOL gr 3; IOM gr 2	-	Syndesmotic fixation + Broström
#74	ATFL gr 2; CFL gr 2	AITFL gr 1; IOL gr 1	Osteochondral ICRS grade 4	Debridement of (O)CLs with BMS
#80	ATFL gr 1; CFL gr 3	AITFL/IOL gr 3; IOM gr 2	-	Syndesmotic fixation
#92	ATFL gr 1; CFL gr 1	AITFL/IOL gr 3; IOM/PITFL gr 2	-	Syndesmotic fixation
#94	ATFL gr 3; CFL gr 2	AITFL/IOL gr 3; IOM gr 2	Cartilage ICRS grade 4	Syndesmotic fixation
#99	ATFL gr 1; CFL gr 2	AITFL/IOL gr 3; IOM/PITFL gr 2	-	Syndesmotic fixation
#109	ATFL gr 3; CFL gr 2	AITFL/IOL/IOM/PITFL gr 2	-	Syndesmotic fixation + Broström
#112	ATFL gr 3; CFL gr 3	AITFL/IOL gr 3; IOM/PITFL gr 2	Cartilage ICRS grade 3D	Syndesmotic fixation + Broström
#130	ATFL gr 1; CFL gr 3	AITFL gr 2	Osteochondral ICRS grade 4	Excision of os trigonum
#141	ATFL gr 3; CFL gr 3	AITFL/IOL gr 2; IOM gr 1	-	Syndesmotic fixation
#165	ATFL gr 3; CFL gr 3	AITFL/IOL/IOM/PITFL gr 2;	-	Syndesmotic fixation + Broström

Clinical description of patients who underwent surgery; *MRI* Magnetic Resonance Imaging; *(O)CLs* cartilage and osteochondral lesions; *ATFL* anterior talofibular ligament; *CFL* calcaneofibular ligament; *AITFL* anterior inferior tibiofibular ligament; *IOL/IOM* Interosseous Ligament/Membrane; *PITFL* Posterior inferior Tibiofibular Ligament; ICRS International Cartilage Repair Society; *BMS* Bone Marrow Stimulation.
